# Assessment of the frequency of sperm annulus defects in a large cohort of patients presenting asthenozoospermia

**DOI:** 10.1186/s12610-015-0026-z

**Published:** 2015-11-15

**Authors:** Thassadite Dirami, Baptiste Rode, Jean-Philippe Wolf, Gérard Gacon, Emmanuel Dulioust, Aminata Touré

**Affiliations:** INSERM U1016, Institut Cochin, Paris, 75014 France; CNRS UMR8104, Paris, 75014 France; Sorbonne Paris Cité, Faculté de Médecine, Université Paris Descartes, Paris, 75014 France; Assistance Publique-Hôpitaux de Paris, GH Cochin Broca Hôtel Dieu, Laboratoire d’Histologie Embryologie - Biologie de la Reproduction, Paris, 75014 France; Department of Genetics, Development and Reproduction, Institut Cochin, INSERM U1016, CNRS UMR8104, Université Paris Descartes, 24 rue du faubourg Saint Jacques, Paris, 75014 France

**Keywords:** Sperm, Flagellum, Annulus, Motility, Asthenozoospermia, SLC26A8, Spermatozoïde, Flagelle, Annulus, Mobilité, Asthénozoospermie, SLC26A8

## Abstract

**Background:**

The annulus is a ring-shaped structure located beneath the plasma membrane that connects the midpiece and the principal piece of mammalian sperm flagellum. It has been suggested that the annulus acts as a morphological organizer, guiding flagellum assembly during spermiogenesis, and as a diffusion barrier, confining proteins to distinct compartments of the flagellum in mature sperm. Previous studies on small cohorts of patients have attempted to correlate annulus defects with the occurrence of human asthenozoospermia. An absence of the annulus has been shown to be frequently associated with asthenozoospermia.

**Findings:**

We tried to obtain a more precise estimate of the frequency of annulus defects, by screening a large cohort of 254 men presenting asthenozoospermia (mean progressive motility of 24 %) by the immunodetection of SLC26A8, a transmembrane protein that has been shown to be specifically localized to the annulus. By contrast to previous reports, our results indicate that annulus defects are associated with asthenozoospermia in only 1.2 % of cases.

**Conclusions:**

We conclude that defects or an absence of the annulus are not frequently associated with asthenozoospermia. The use of annulus defects as a diagnostic endpoint in patients is therefore not appropriate.

## Resume

**Introduction:**

L’annulus (Anneau de Jensen) est localisé à la jonction de la pièce intermédiaire et de la pièce principale du flagelle des spermatozoïdes de mammifères. Sa fonction reste encore mal établie mais il est suggéré qu’il puisse être essentiel à l’assemblage du flagelle et à la compartimentation des protéines le long du flagelle. Des études précédentes réalisées sur des petites cohortes de sujets asthénozoospermiques ont mis en évidence des défauts fréquents de l’annulus sur les spermatozoïdes de ces sujets.

**Resultats:**

Afin de mieux estimer la fréquence des défauts de l’annulus chez les sujets asthénozoospermiques, nous avons analysé une cohorte de 254 sujets asthénozoospermiques (mobilité moyenne 24 %) par immunodétection de l’annulus, en utilisant un anticorps spécifique de la protéine SLC26A8, un constituant établi de l’annulus. Nos résultats indiquent que les défauts ou absence de l’annulus ne sont retrouvés qu’à une fréquence de 1.2 %.

**Conclusions:**

A partir de cette étude réalisée sur le plus grand effectif de sujets asthénozoospermiques à ce jour, nous pouvons conclure que les défauts ou l’absence d’annulus ne sont pas fréquemment associés à l’asthénozoospermie modérée; l’utilisation de l’annulus comme outil de diagnostic de ce type d’asthénozoospermies, comme initialement suggéré, ne nous semble donc pas applicable.

## Background

**Fig. 1:**
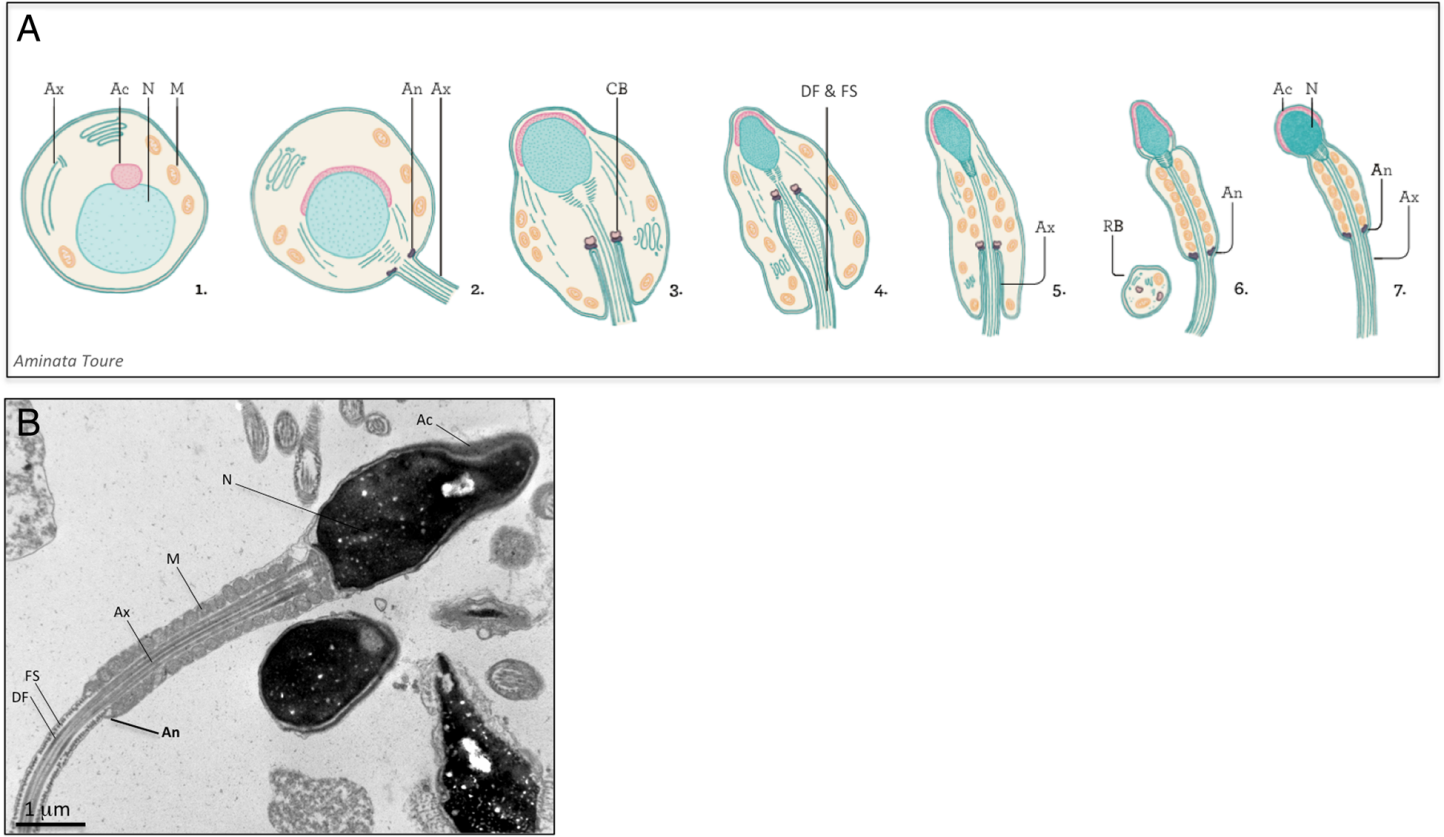
**a** Flagellum assembly during sperm terminal differentiation in mammals. *Scheme from Aminata Toure.* Following meiosis, the spermatids undergo a set of morphological changes that specify the flagellum and the acrosome (Ac), required for motility and interaction with the oocyte, respectively. The acrosome (Ac) is formed by the fusion of vesicles from the Golgi apparatus, and the nucleus (N) is highly condensed by the germ cell-specific histones and protamines (steps 1–3). The flagellum is formed by the assembly of the microtubules, which constitute the axoneme (Ax) and the periaxonemal structures, the dense fibers (DF) and fibrous sheath (FS). The annulus (An) is assembled in the cytoplasm, at very early stages of flagellum assembly (step 2). It then associates with another electron-dense structure, the chromatoid body (CB), which consists mostly of RNA and RNA-binding proteins (step 3). During the extension of the flagellum,, the mitochondria (M) align along the axoneme (Ax) and the excess cytoplasm is removed as residual bodies (RB). The annulus (An) then moves towards its final position at the junction of the midpiece and the principal piece (steps 4–7). *Ac: acrosome; An: annulus; Ax: axoneme; CB: chromatoid body; RB: residual body; DF: dense fibers; FS: fibrous sheath; M: mitochondria; N: nucleus.* **b** Electron micrograph of human spermatozoa. *Image from Aminata Touré & Alain Schmitt.* Human spermatozoa with the head on the right, and the flagellum on the left. The flagellum is divided into two main compartments: the midpiece, which comprises the mitochondrial sheath, and the principal piece, characterized by the presence of a fibrous sheath surrounding the axoneme. The annulus is distinguishable at the junction of the midpiece and principal piece, as a fine electron-dense structure apposed to the plasma membrane. Bar: 1 μm. *Ac: acrosome; An: annulus; Ax: axoneme; CB: chromatoid body; RB: residual body; DF: dense fibers; FS: fibrous sheath; M: mitochondria; N: nucleus*

The annulus is a ring-shaped structure located beneath the plasma membrane that connects the midpiece and the principal piece of mature sperm flagellum. It is formed in the round spermatids at very early stages of flagellum assembly, concomitant with nucleus condensation and the development of the acrosome (Fig. 1). It was long thought, based on the spatiotemporal distribution of this structure during sperm flagellum elongation, that it might behave as a morphological organizer, guiding the growth of the flagellum and the alignment of the mitochondria along the axoneme [1]. In addition, its position between the two major compartments of the flagellum in mature spermatozoa suggested that it might act as a diffusion barrier confining proteins to particular compartments of the sperm tail [2, 3]. Strong evidence to support these two hypotheses has been provided by the recent identification of protein components of the annulus and the characterization of mice in which the corresponding genes have been knocked out [4–8]. However, the precise molecular mechanisms underlying the biogenesis and function of the annulus remain unclear.

Only a few proteins have been shown to localize to the annulus. The first to be identified were proteins of the Septin family (Septins 1, 4, 6, 7 and 12), small G proteins forming homo- and heterofilaments in various cell types and compartments [4, 5, 7]. *Septin 4* invalidation in the mouse resulted in male infertility due to total asthenozoospermia, and morphological defects of the flagellum, including an absence of the annulus, a bending of the flagellum and an abnormal arrangement of the mitochondria in the midpiece. Annulus defects were also observed in *Septin 12* mutant mice [4, 5, 7, 9]. DNAJB13, a member of the HSP40 co-chaperone family, and SLC26A8 (TAT1; testis anion transporter 1), a sperm-specific anion transporter, were subsequently also localized to the annulus [6, 10]. DNAJB13 is located in the radial spokes of the (9 + 2) axoneme in mouse sperm and in the *Chlamydomonas reinhardtii* flagellum [10–13]. It has been reported to interact physically with Septin 4 at the annulus during spermiogenesis. SLC26A8 is a sperm-specific member of the SLC26 (solute-linked carrier 26) family of anion exchangers that has been shown to be restricted to the sperm annulus and head equatorial segment in humans and mice [6, 14]. Interestingly, *Slc26a8* inactivation in the mouse results in a near-phenocopy of the Septin 4-null mouse, suggesting that this protein is also essential for annulus integrity, sperm flagellum morphology and motility [6]. In *Slc26a8* mice, electron microscopy studies have shown the sperm annulus to be present but markedly atrophic and detached from the plasma membrane. This transmembrane protein is thought to play a structural role, anchoring the cytoplasmic components of the annulus to the plasma membrane, in addition to its regulatory function in mediating the anion fluxes required to induce sperm motility and capacitation [15]. A few enzymes, such as the soluble adenylate cyclase (sAC) activated during sperm motility, and the vitamin D-metabolizing enzyme CYP24A1 have been found to localize at the annulus [16, 17]. The reasons for this restriction to the annulus, as opposed to the location along the length of the principal piece observed for other enzymes required for sperm motility and function, remain unclear.

Despite these recent findings in mice, the importance of the annulus for mammalian sperm flagellum structure and motility has not been clearly established. Several studies have attempted to resolve these questions by analysing the correlation between annulus defects and the occurrence of human asthenozoospermia. A first study on a cohort of 20 Japanese patients with asthenozoospermia indicated that 15 % of these patients had no annulus, leading the authors to claim that an absence of the annulus could be used as a diagnostic marker for a subset of patients with asthenozoospermia [5]. A larger cohort of 108 Japanese men with normal and low sperm motility was then analysed and a similar frequency (13 %) was reported for the absence of the annulus [18]. We studied a cohort of 75 asthenozoospermic patients but observed an absence of the annulus in only one of these patients, resulting in a much lower frequency (1.2 %) [19]. We assumed that this discrepancy between our results and those of previous studies reflected ethnic differences or the small sizes of the cohorts studied to date. In this study, we analysed a new, larger cohort of asthenozoospermic patients with precisely characterized phenotypes, to determine the frequency of annulus defects in asthenozoospermia more accurately. We screened sperm preparations from 254 additional asthenozoospermic patients for the presence of the annulus, by an immunodetection method, with specific antibodies directed against the SLC26A8 protein, a previously described component of the annulus that can be used as a marker of this structure.

## Materials and methods

### Patients

The study was conducted in accordance with hospital ethics guidelines. We studied semen samples from patients giving informed consent, for whom semen analysis was performed between March 2008 and July 2010 at the Laboratory of Reproductive Biology of Cochin Hospital (Paris, France). Approval was obtained from the ethics evaluation committee of the ‘Institut National de la Santé et de la Recherche Médicale’ (authorization number 01–013) and the ‘Comité de Protection des Personnes CPP Ile de France III’ (authorization number Sc-2748).

**Fig. 2:**
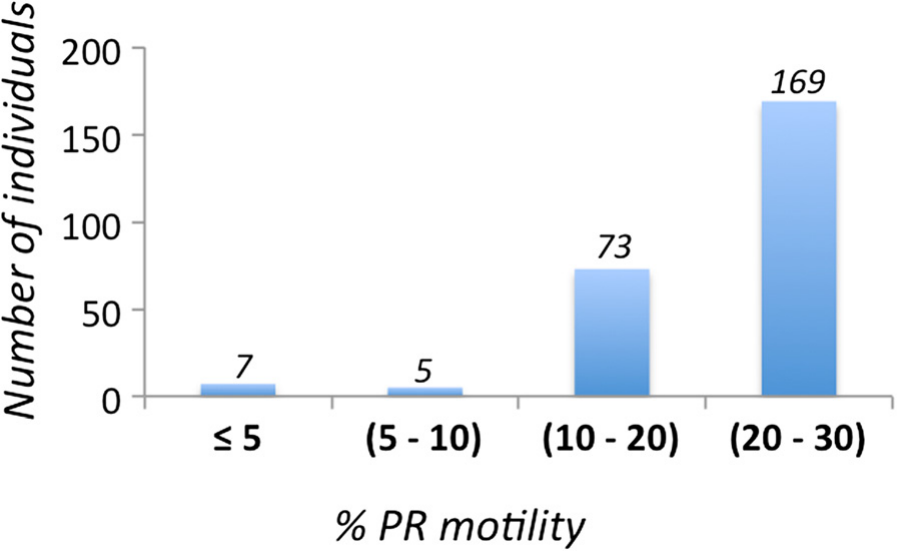
Sperm progressive motility in the cohort. Histogram showing the distribution of progressive motility (PR) values for the 254 individuals included in the cohort. Most of the patients (242) have a PR value above 10 %

The criteria for inclusion in the study were asthenozoospermia, defined as a percentage of progressive sperm (PR; a + b type motility) in the ejaculate of less than 32 %, according to the reference values established by the World Health Organization [20], and a percentage of viable sperm above 50 %. The mean sperm parameters of the cohort were as follows: progressive motility, PR, 24 % (0 – 30 %); sperm vitality, 71 % (50 – 89 %); sperm concentration, 81 million spermatozoa per ml (12.5 – 281.8 million); ejaculate volume, 4 ml (0.8 – 9.8 ml). Most of the patients (*n* = 242) had a PR greater than 10 % (Fig. 2).

### Human sperm evaluation

Semen samples were obtained by masturbation after a period of three to five days of abstinence. The samples were incubated at 37 °C for 30 minutes, for liquefaction. Ejaculate volume and pH, sperm concentration, vitality, morphology and motility were evaluated according to World Health Organization (WHO) guidelines. Sperm motility was assessed by computer-aided sperm analysis (CASA) and sperm were classified as progressive (a- and b- types), non-progressive (c-type) and immotile (d-type). Sperm vitality was assessed by eosin staining and sperm morphology, by Giemsa staining. The semen samples studied here correspond to what was left of the original sample after all the clinical evaluations had been performed. These samples were stored frozen until their use for the immunodetection assay.

### Immunodetection assay

We spread 10 μl of frozen sperm onto a Superfrost Plus slide (Menzel Glasbearbeitungswerk, GmbH & Co. KG, Braunschweig, Germany). The sperm was fixed by incubation with cold methanol/acetone (3/1 ratio v/v) for 10 minutes. The slides were treated with 0.2 % Triton in PBS for permeabilization and then blocked by incubation in 1 % BSA for 1 hour. They were then incubated with primary antibodies for 2 hours at room temperature and then secondary antibodies for one hour at room temperature. The slides were mounted in Vectashield medium (Vector Laboratories, Burlingame, USA) supplemented with 0.5 mg/ml DAPI. Slides were analyzed with a Zeiss Axiophot epifluorescence microscope. Digital images were acquired with a cooled charge-coupled device (CCD) camera (Hamamatsu Co. Japan), under identical instrument settings, with MetaMorph® software (Molecular Devices, Inc. USA).

Primary antibodies: The L2CL4 antibody was raised in rabbit, against human SLC26A8 amino acids 664–970 [21]; the SE5362 antibody was raised in rabbit, against amino acids 1–15 and amino acids 955–970 of human SLC26A8, with purification against both peptides (Eurogentec) [14]; the anti-SEPTIN 4 antibody H-120 was obtained from Santa Cruz Biotechnology. Mitotracker Red 580 was used for midpiece staining.

## Results

We performed immunodetection assays on sperm preparations for all 254 men, using the SE5362 antibody, which detects the SLC26A8 protein at both the annulus and the equatorial segment of human and mouse spermatozoa [14]. In cases of abnormal staining for SLC26A8, the results were checked by staining with another antibody, L2CL4, which detects SLC26A8 at the annulus [21], and double-staining of the midpiece with Mitotracker Red 580 (Molecular Probes). Three of the 254 asthenozoospermic patients screened, I1, I2 and I3, displayed a lack of annulus staining, with both antibodies, with no impairment of staining for the equatorial segment. The main semen parameters of individuals I1, I2 and I3 are presented in Table 1.

**Table 1:** Semen characteristics of individuals I1, I2 and I3 identified as displaying no SLC26A8 staining of the sperm annulus. Values were compared with the lower reference limits established by the World Health Organization [20]

Individual				Lower reference limits (WHO, 2010)
	I1	I2	I3	
Age	40	38	35	
Volume of ejaculate (ml)	6	2.4	1.4	1.5
pH	7.9	8.3	7.7	7.2
Total sperm count (10^6^ /ejaculate)	186	76.8	182	39
Progressive motility, PR (%)	20	30	30	32
Viability (%)	59	79	75	58
Sperm morphology				
Typical forms (%)	12	3	8	-
Flagellar abnormalities (%)	17	15	15	-

Patient I1, aged 40, was consulting for medically assisted procreation because his wife was infected with HIV and presented bilateral tubal obstruction. Patient I1 had no specific history of genital disease, but he displayed bilateral varicocele and obesity. Semen analysis showed a moderately low progressive motility (PR 20 %) and percentage of typical forms, following no particular pattern. Selection by centrifugation through a density gradient increased PR to 70 %, with 40 % rapid spermatozoa, and increased the percentage of typical forms to 19 %. A pregnancy was obtained by *in vitro* fertilization and the couple had a healthy child.

Patient I2 was 38 years old and had no previous medical or genital health history. He and his partner had been trying to have children for six years and had suffered a miscarriage two years previously. Semen analysis showed PR to be almost normal (PR 30 %). Only 5 % of the spermatozoa were considered to be rapid, but semen viscosity was high and, after selection, 70 % of the selected spermatozoa were rapidly motile. The percentage of typical spermatozoa was very low, but most of the abnormalities observed concerned the acrosomal region of the sperm head.

Patient I3 was 35 years old. His partner had had two natural pregnancies in the two years preceding semen analysis at our center: the first was an ectopic pregnancy, and the second ended in miscarriage. No other information about fertility or medical history was available. The couple was followed elsewhere, but we were informed that they achieved a natural pregnancy, with a healthy child born the following year. The semen parameters were close to normal values (PR 30 %), except for sperm morphology, but most of the morphological abnormalities observed concerned the sperm head and followed no particular pattern.

**Fig. 3:**
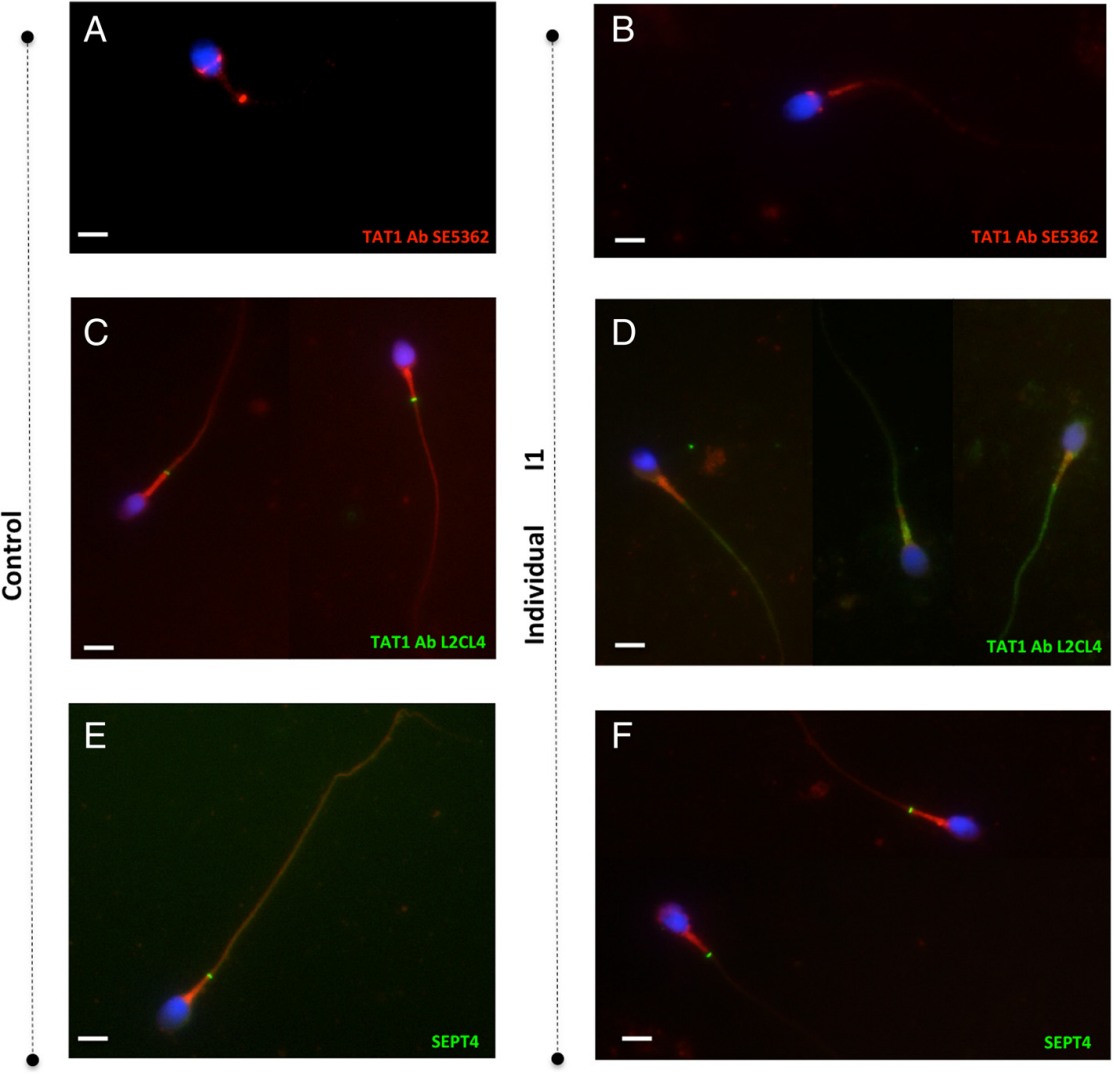
Immunodetection of SLC26A8 and SEPTIN 4 on sperm preparations from individual I1. **a, c** and **e** Control semen and **b, d** and **f** patient semen. Antibodies SE5362 and L2CL4, directed against SLC26A8, label the annulus of control spermatozoa, but not of spermatozoa from individual I1. The SE5362 antibody correctly detects SLC26A8 at the equatorial segment in spermatozoa from both control and individual I1. SEPTIN 4 is detected at the annulus in spermatozoa from both the control and individual I1. Mitotracker Red 580 was used for MP staining. Bars: 5 μm

An analysis of sperm from individual I1 with antibody SE5362 showed that the SLC26A8 signal was spread out along the flagellum, rather than being located in the annulus as in control sperm (Fig. 3a, b), whereas detection in the equatorial segment was normal. This result was confirmed by staining with the L2CL4 antibody, which showed that SLC26A8 was absent from the annulus (Fig. 3c, d). A signal for SEPTIN 4 (SEPT4) was observed at the annulus in immunodetection experiments, suggesting that the annulus was incomplete rather than absent (Fig. 3e, f). Sperm from individuals I2 and I3 displayed no SLC26A8 signal at the annulus or the equatorial segment (data not shown). It was not possible to perform immunodetection with SEPTIN 4 (SEPT4) for these individuals. The identification of an abnormal pattern at the annulus in immunodetection studies for these individuals suggested that the annulus might be modified or missing from their spermatozoa. Unfortunately, too small volume of semen remained for electron microscopy and we were therefore unable to carry out an ultrastructure analysis.

We had previously screened this cohort of patients by genomic amplification and sequencing of sperm DNA. We found mutations of *SLC26A8* (MIM 608480) responsible for SLC26A8 protein instability and proteasomal degradation in three different patients from those we present here [22]. We therefore investigated whether the observed absence of the SLC26A8 protein at the sperm annulus was due to mutations of *SLC26A8*. An analysis of the coding regions of *SLC26A8* in individuals I1 and I2 identified only the most frequently described sequence polymorphisms (I2: p.M73V and p.V639I). We therefore concluded that the absence of the SLC26A8 protein at the annulus was probably due to a defective structure of the annulus, preventing the correct localization of the protein, consistent with the staining for SLC26A8 observed along the flagellum.

In total we found that only three of the 254 asthenozoospermic individuals in the cohort studied here (1.18 %) had a potentially incomplete or absent annulus. In a previous study of 75 asthenozoospermic patients, only one individual with a complete lack of the sperm annulus was identified by the immunodetection of SLC26A8 and SEPTIN, with confirmation by electron microscopy [19]. If we consider all the individuals included in these two studies together, 329 patients, then the overall frequency of annulus defects in our population of asthenozoospermic patients can be estimated at 1.21 %.

## Discussion

In this study, we investigated the integrity of the annulus on sperm preparations from 254 patients presenting proven asthenozoospermia, according to the current WHO criteria (progressive motility < 32 %). By combining the data obtained in this study with those for 75 asthenozoospermic patients from a previous study [19] we were able to analyse 329 individuals in total. This cohort is the largest cohort of individuals studied to date in investigations of the association between annulus abnormalities and the occurrence of asthenozoospermia. Our data indicate that defects or an absence of the annulus are rarely detected, with an estimated frequency of only 1.2 % in human patients with asthenozoospermia and a progressive motility of more than 10 %. Our findings differ from the results initially published for the analysis of smaller cohorts of Japanese asthenozoospermic individuals, which indicated a high frequency of annulus defects in this population (13 to 15 %). The difference between our results and those of these previous studies may reflect differences in ethnic origin, as the patients of the cohort studied here were mostly Caucasian men; in addition, SLC26A8 defects may constitute only a subset of the annulus defects present, as SEPTIN proteins form the core of the annulus. However, our data and published findings do not support these two hypotheses. Hence Hosseinifar and colleagues recently analyzed sperm from a cohort of 100 asthenozoospermic Iranian men. They carried out immunodetection for the SEPTIN 4 and SEPTIN 7 proteins on sperm samples from their patients and found only one individual lacking the annulus, as confirmed by electron microscopy. Their conclusion for the Iranian population was therefore similar to that reported here: a very low frequency of annulus defects in the population studied [23]. In addition, our unpublished data for analyses of *Slc26a8* and *Septin4* knockout mice (provided by H. Kissel & H. Steller) indicate that the lack of Septin 4 prevents the localization of Slc26a8 to the annulus. Thus any annulus defect due to the absence of Septin 4 should have been identified by staining with the SLC26A8 antibody. Based on all these findings, we conclude that the integrity of the annulus is not a relevant diagnostic endpoint for screening and classifying asthenozoospermic patients.

Overall, caution is currently required when drawing conclusions about the possible function and causality/pathogenicity to be attributed to the annulus. The molecular mechanisms by which this intriguing structure is assembled and functions remain unclear, in both humans and mice; the possible role of annulus abnormalities in human asthenozoospermia also remains to be determined. As several lines of evidence suggest that the annulus is involved in guiding flagellum assembly (in particular, in midpiece arrangement during sperm terminal differentiation), annulus defects may be be the cause of asthenozoospermia due to abnormal flagellum biogenesis in humans, a phenotype, which would be however distinct from that of Septin4 and Slc26a8 invalidation in mice. This phenotype, previously described as dysplasia of the fibrous sheath [24–27] and, more recently, as multiple morphological abnormalities of the flagella (MMAF) [28], consists of a lack of flagellum assembly and the presence of short, bent and coiled flagella of irregular thickness. Further studies of the annulus in sperm from MMAF patients are required to address this aspect.

## Abbreviations

PR: progressive motility
SLC26A8: solute-linked carrier 26A8
SEPT: SEPTIN
MMAF: multiple morphological abnormalities of the flagellum
